# Legacy of the Lost and Pressure of the Present: Malagasy Plant Seeds Retain Megafauna Dispersal Signatures but Downsize Under Human Pressure

**DOI:** 10.1111/ele.70205

**Published:** 2025-09-10

**Authors:** Yuanshu Pu, Alexander Zizka, Renske E. Onstein

**Affiliations:** ^1^ Faculty of Life Sciences University of Leipzig Leipzig Germany; ^2^ German Centre for Integrative Biodiversity Research (iDiv) Halle‐Jena‐Leipzig Leipzig Germany; ^3^ Department of Biology Philipps‐University Marburg Marburg Germany; ^4^ Naturalis Biodiversity Centre Leiden the Netherlands; ^5^ Institute of Biology Leiden Leiden University Leiden the Netherlands

**Keywords:** defaunation, endozoochory, frugivory, Madagascar, megafauna extinction, seed dispersal, structural equation model, trait matching

## Abstract

Ongoing declines of large‐bodied frugivores limit the dispersal of large‐seeded plants, contributing to their (local) demise and ‘downsizing’ of seeds across assemblages. However, the extent to which human pressure leads to contemporary seed downsizing, and whether extinct megafrugivores have left imprints on seed size, remains unclear. Here, we integrate trait and distribution data for 2852 endozoochorous plant species, 48 extant and 15 extinct frugivore species across 361 assemblages on Madagascar. Using structural equation models, we show that assemblages with higher human footprint, a cumulative index of human pressure, have smaller maximum seed sizes, especially through downsizing of extant frugivores. Furthermore, among assemblages with ‘mega‐seeded’ plants (i.e., seeds that cannot be swallowed by any extant Malagasy frugivore), larger seed sizes are associated with larger past megafrugivores, reflecting the legacy of past interactions. Human‐driven seed downsizing highlights broader implications in erosions of important ecosystem functions such as forest carbon storage.

## Graphical abstract

Using structural equation models, we show that Malagasy assemblages with higher human pressure have smaller maximum seed sizes, especially through downsizing of extant frugivores. Furthermore, among assemblages with ‘mega‐seeded’ plants (i.e., seeds that cannot be swallowed by any extant Malagasy frugivore), larger seed sizes are associated with larger past megafrugivores, reflecting the legacy of past interactions. Human‐driven seed downsizing highlights broader implications in erosions of important ecosystem functions such as forest carbon storage.

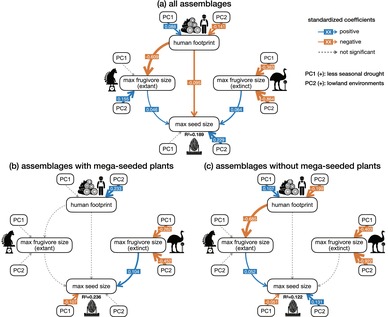

## 1 Introduction

Co‐evolution and niche tracking between mutualistic plants and vertebrates shape the composition of contemporary local assemblages and their interaction networks (Chagnon et al. 2015; Dalsgaard et al. 2021; Durand‐Bessart et al. 2023). Additionally, the impact of climate and human activities on the spatial distribution of species modifies assemblage compositions (Barnagaud et al. 2017; Joswig et al. 2021). However, the respective magnitude of past and present mutualistic interactions and how they have shaped contemporary assemblage trait structures remains unclear.

Endozoochory is the mutualism between fruit‐producing plants and fruit‐eating animals (frugivores, hereafter), in which frugivores consume fleshy fruits and disperse the contained seeds after gut passage. In this mutualism, the size of the ingested seeds is constrained by the frugivore's gape width, which aligns with the frugivore's body mass (Burns 2013; Lord 2004; Wheelwright 1985). Larger seeds benefit from dispersal by larger‐bodied animals, with dispersal over longer distances, higher seedling survival rates, reduced resource competition with relatives, and increased opportunities for successful colonisation of new habitats (Chapman 2005; Onstein et al. 2019; Pires et al. 2018; Wotton and Kelly 2012). However, larger‐seeded plants are restricted to fewer frugivore species that can ingest their seeds.

Spatial patterns of ‘trait matching’ (i.e., a positive correlation) between frugivore traits (e.g., gape width or body mass) and plant traits (e.g., fruit width or seed width) have been discovered worldwide (Dehling et al. 2014; Mack 1993; McFadden et al. 2022). However, such spatial correlation can be disrupted (Lim et al. 2020; Méndez et al. 2022) as extinctions of large‐bodied animals reduce the maximum body size in many assemblages worldwide (i.e., ‘downsizing’; Dirzo et al. 2014; Young et al. 2016). Consequently, seed dispersal limitation following frugivore downsizing can lead to structural (e.g., secondary plant extinctions) and functional (e.g., loss of long distance dispersal) decay in plant assemblages (Donoso et al. 2020; Fricke et al. 2022). However, it remains unclear whether it could ultimately lead to changes in assemblage‐wide trait structures, such as seed size.

Frugivore downsizing has been particularly severe in Madagascar, where all animals with a body mass over 10 kg rapidly went extinct ca. 1000 years ago (Crowley 2010). Additionally, 94% of the remaining lemur species, the largest and most important extant frugivore guild in Madagascar, are threatened with extinction (Albert‐Daviaud et al. 2018; IUCN 2022; Razafindratsima 2015). As more than 30% of plant species in Madagascar depend on endozoochorous seed dispersal (3324 out of 9434 endemic species; Albert‐Daviaud et al. 2018; Antonelli et al. 2022), the loss of frugivorous lemurs may have detrimental consequences for the Malagasy flora.

Interestingly, the seeds of some Malagasy plant species, for example, *Borassus madagascariensis* (Arecaceae, seed width ca. 68 mm; species nomenclature follows Plants of the World Online (POWO 2025), hereafter) and *Tsebona macrantha* (Sapotaceae, seed width ca. 38 mm), are larger than the gape width of any extant frugivorous lemur. The largest seeds recorded to be dispersed by extant frugivores are 24.6 mm wide, from *Canarium madagascariense* (Burseraceae; Albert‐Daviaud et al. 2020; Razafindratsima and Martinez 2012), suggesting that plants with seeds larger than this threshold are not effectively dispersed through endozoochory anymore. These species (mega‐seeded plants, hereafter) are therefore ‘anachronistic’—that is, they possess large fruits and seeds that seem adapted to past biotic interactions but maladapted in current ecosystems (Janzen and Martin 1982). It is unclear how these mega‐seeded plants have persisted in ecosystems since the extinction of their primary megafaunal seed dispersers (megafrugivores, hereafter) and whether their distributions still reflect historical trait matching with now‐extinct megafrugivores (Heinen et al. 2023; Méndez et al. 2022).

In addition to seed dispersal limitation due to human‐driven frugivore downsizing, endozoochorous plants, particularly those with large seeds, also face direct human pressure. Due to allometric scaling, large seeds are often found in large trees (Leishman et al. 1995; Moles et al. 2005), which are especially susceptible to human activities such as timber logging (Asner et al. 2009). In Madagascar, over 44% of the forest has been cleared since the 1950s by humans, for instance for slash‐and‐burn agriculture and pasture (Scales 2011; Vieilledent et al. 2018). Moreover, the slow growth and recruitment rates of large trees with large seeds further constrain their recovery post disturbance (Barczyk et al. 2024; Moles and Westoby 2006; Osuri and Sankaran 2016; Palma et al. 2021). Despite these theoretical expectations, it remains unknown whether human pressure has affected the distribution of larger‐seeded plants in Madagascar.

In this study, we take an assemblage‐wide approach, assuming potential interactions among co‐occurring species, to disentangle the direct and indirect effects of human footprint, a cumulative index of human pressure, on seed size in endozoochorous plants in Madagascar, considering the contemporary and past distributions of (mega‐)frugivores. First, we hypothesise (H1) that human pressure leads to downsizing of seeds in assemblages, both directly (e.g., due to selective logging of large‐seeded trees) and indirectly, via downsizing of extant frugivore assemblages (e.g., due to hunting; ‘downsizing‐effect hypothesis’). Accordingly, we expect that maximum frugivore and seed sizes in assemblages decrease with increasing human footprint, and that there is a positive relationship between frugivore body mass and seed size across assemblages (reflecting spatial trait matching). Second, we hypothesise (H2) that the occurrence of anachronistic mega‐seeded plants is spatially matched with the previous occurrence of now extinct megafrugivores, but not with the occurrence of extant frugivores (‘legacy‐effect hypothesis’). Hence, we expect that among assemblages with mega‐seeded plants, seeds are larger in assemblages where larger (now extinct) megafrugivores occurred. In contrast, among assemblages without mega‐seeded plants, we expect that seeds are larger in assemblages with larger extant frugivores. By testing these hypotheses, we provide insights into how past megafrugivores and present human pressure jointly shape contemporary seed size distributions, with broader implications for the potential erosion of ecosystem functions due to seed downsizing in tropical ecosystems.

## 2 Material and Methods

### 2.1 Endozoochorous Plant Species

We used the species checklist of endemic endozoochorous seed plants in Madagascar from Albert‐Daviaud et al. (2020; Table S1). Endozoochory was defined by the presence of edible rewards such as fruit pulp or an aril (van der Pijl 1972). Out of 3241 endozoochorous plant species, we obtained occurrence data for 2852 species from Ralimanana et al. (2022), based on validated records from the iNaturalist research‐grade observations and specimens from the Missouri Botanical Garden, accessed via the Global Biodiversity Information Facility (GBIF.org 2020a, 2020b). The raw records were filtered by excluding records from centroids of Madagascar and its provinces, the capital city Antananarivo, and registered research institutions, using *CoordinateCleaner* (Zizka et al. 2019).

We obtained seed widths (the second largest dimension of a seed, which is the limiting axis for animals to ingest a seed) for all endemic endozoochorous plants in Madagascar from Albert‐Daviaud et al. (2020; Table S1). This dataset included both measured and imputed values: seed widths of 1207 species (42.3% out of 2852 species) were collected from botanical descriptions, databases, specimens from the Royal Botanic Gardens, Kew and the Muséum National d'Histoire Naturelle, Paris (Albert‐Daviaud et al. 2020). For the remaining 1645 species (57.7% out of 2852 species), seed widths were imputed by Albert‐Daviaud et al. (2018, 2020) using multivariate imputation by chained equations (MICE; van Buuren and Groothuis‐Oudshoorn 2011). To account for imputation uncertainty, 20 independent imputed datasets were generated based on eleven traits related to fruit or seed morphology, life form, bioclimate and vegetation formation of 8788 species across the Malagasy flora (Albert‐Daviaud et al. 2020). We conducted all subsequent analyses separately on each of the 20 imputed datasets and pooled the results using Rubin's rules (Rubin 1987). To test the robustness of our findings to the data imputation, we repeated all analyses using only directly measured seed widths, excluding imputed values. Finally, among all endemic endozoochorous plants in Madagascar, 15 species (none with imputed seed width; Table 1) had seeds that were wider than 24.6 mm—the width of the largest seed (*Canarium madagascariense*) recorded to be dispersed by any extant Malagasy frugivore (Albert‐Daviaud et al. 2020; Razafindratsima and Martinez 2012). These species were considered ‘anachronistic’ and classified as mega‐seeded plants in our analyses.

**TABLE 1:** List of 15 mega‐seeded endozoochorous plant species.

Species	Family	Seed width (mm)	IUCN red list status
*Borassus madagascariensis*	Arecaceae	67,5	Endangered
*Tsebona macrantha*	Sapotaceae	37,5	Near threatened
*Orania longisquama*	Arecaceae	37,0	Least concern
*Mauloutchia heckelii*	Myristicaceae	35,0	Endangered
*Antiaris madagascariensis*	Moraceae	32,0	NA
*Satranala decussilvae*	Arecaceae	32,0	Endangered
*Lemurophoenix halleuxii*	Arecaceae	31,5	Endangered
*Noronhia populifolia*	Oleaceae	28,4	Critically endangered
*Beilschmiedia cryptocaryoides*	Lauraceae	27,0	Data deficient
*Magnistipula cerebriformis*	Chrysobalanaceae	27,0	Vulnerable
*Gluta tourtour*	Anacardiaceae	26,5	Vulnerable
*Beilschmiedia velutina*	Lauraceae	26,2	Least concern
*Mammea sessiliflora*	Calophyllaceae	25,1	Least concern
*Antiaris toxicaria*	Moraceae	25,0	Least concern
*Euphorbia mandravioky*	Euphorbiaceae	25,0	Vulnerable

*Note:* Mega‐seeded plant species are defined as those species that have seeds that were wider than 24.6 mm—the width of the largest seed (*Canarium madagascariense*) recorded to be ingested and dispersed by any extant Malagasy frugivore (Albert‐Daviaud et al. 2020; Razafindratsima and Martinez 2012). The International Union for Conservation of Nature (IUCN) Red List status data is collected from IUCN (2022). Species nomenclature follows Plants of the World Online (POWO 2025).

### 2.2 Extant and Extinct Frugivores

We considered all extant vertebrate species on Madagascar with diets consisting of at least 50% of fruits as ‘frugivores’ (48 species in total). We used this threshold as these animals are critical for endozoochorous dispersal of large‐seeded plants (Fuzessy et al. 2018). Additionally, this strict threshold was necessary for reducing noise in our coexistence‐based method, as the traits and distributions of facultative frugivores are likely influenced by broader factors beyond seed dispersal (Eppley et al. 2020). For extant frugivores, we used the frugivory index data from EltonTraits 1.0 (Wilman et al. 2014) and Galán‐Acedo et al. (2019). Sixteen species had no dietary data available, for which we inferred the most probable dietary group based on information from other species within the same genus. The final dataset included 28 primates, 3 bats, 11 birds and 6 rodents (Table S2). We obtained distribution ranges for these species from the International Union for Conservation of Nature Red List, ver. 2022–2 (IUCN 2022), and body mass data by calculating the average values across available species‐level data from Wilman et al. (2014), Galán‐Acedo et al. (2019) and Razafindratsima, Gentles, et al. (2018); (Table S2).

As extinct megafrugivores in Madagascar, we included four elephant bird species, nine giant lemur species, and two giant tortoise species, following Méndez et al. (2022, 2024), (Table S2). We obtained historical distribution maps for these species from Méndez et al. (2022) and Pedrono et al. (2013), which were reconstructed using MInOSSE (Carotenuto et al. 2020). This model‐based method uses the distributions of coeval fossil species (i.e., species that existed at the same time) instead of relying on environmental predictors, and therefore has been shown to perform well, especially when the fossil record of the target species is scarce (Carotenuto et al. 2020). We obtained body mass data of extinct megafrugivores from Jungers et al. (2008), Hansford and Turvey (2018), Hansen et al. (2008) and the Turtle Taxonomy Working Group (2021), where body masses were inferred from bone and carapace measurements (Table S2).

### 2.3 Assemblage‐Level Data

We rasterized the terrestrial area of Madagascar into 649 grid cells of 30 km x 30 km, each representing an assemblage of co‐occurring endozoochorous plant and frugivore species (Table S3). This resolution was selected to balance between spatial resolution and data availability. To assess the sensitivity of our analyses to grid size, we repeated the rasterization and all subsequent steps using alternative resolutions of 15 × 15 km and 45 × 45 km. Species occurrence and range maps were then intersected with the assemblages to generate presence‐absence matrices for all plant and frugivore species. Next, to capture the upper range of trait values in an assemblage while reducing the influence of extreme outliers, we calculated the 95th percentile maximum plant seed width (maximum seed width, hereafter) and the 95th percentile maximum frugivore body mass (maximum body mass, hereafter) for each assemblage. These upper‐tail metrics were particularly relevant to our hypotheses, as they represent the species most likely involved in interactions between large seeds and large‐bodied frugivores, which were expected to be most affected by human pressure and extinctions of larger‐bodied frugivores.

We excluded assemblages on the coast with less than 50% terrestrial area, assemblages that lacked extinct frugivores, assemblages with fewer than three extant frugivore species, and assemblages with fewer than three endozoochorous plant species (361 out of 649 assemblages remained for the 30 km grid; 752 out of 2446 assemblages remained for the 15 km grid; 199 out of 306 assemblages remained for the 45 km grid). This allowed us to focus on patterns that emerged from mutualistic interaction‐related processes as outlined in our hypotheses, while avoiding biased results due to outlier assemblages with few species.

### 2.4 Human Footprint and Environmental Factors

We obtained the human footprint map of 2009 from Venter et al. (2016), which compiled and quantified different human stressors related to land use transformation (built environment, crop and pasture lands, night‐time lights), transportation infrastructure (roads, railways and navigable waterways) and human population density. As the human footprint map had a finer spatial scale (1 × 1 km) than our assemblage grid, we intersected it with our assemblages and calculated the average value of the cumulative human footprint for each assemblage.

While our focus was on the effects of human footprint and frugivore body mass on seed width, we accounted for environmental factors to avoid confounding effects. Environmental conditions, such as higher temperature and precipitation, can independently promote larger seeds by providing greater energy and resource availability (Moles et al. 2007), or promote smaller mammalian frugivores due to heat conservation mechanisms (Rodríguez et al. 2008). Without considering these factors, the relationship between seed width and frugivore body mass could be absent due to confounding environmental effects, or conversely, observed relationships could be misattributed to mutualistic interactions rather than shared environmental drivers. We obtained environmental data from MadaClim (https://madaclim.cirad.fr/). For each assemblage, we calculated mean values of 26 variables related to temperature, precipitation, topography, solar radiation, water deficiency and percentage of forest cover (for a complete list of variables, see Table S4). Then, to reduce the dimensionality of the dataset while capturing the most variance, we normalised and scaled all variables and conducted principal component analysis (PCA). The first two principal components (39.63% variance explained by PC1, 31.62% by PC2) were used in subsequent analyses.

### 2.5 Structural Equation Modelling

**FIGURE 1:**
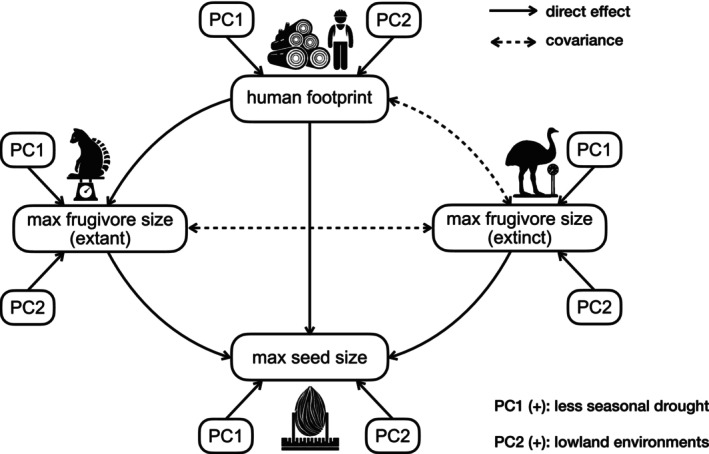
A priori structural equation model for testing the relationships between seed size, frugivore size, human footprint and environmental factors in Malagasy plant assemblages. Solid directional arrows indicate direct effects; dashed bidirectional arrows indicate covariance allowed in the model. Effects of the first two principal components of environmental factors on seed size, frugivore size, and human footprint are shown next to each variable. Max seed size = 95th percentile maximum seed width in the assemblage; max frugivore size (extant) = 95th percentile maximum body mass of extant frugivores in the assemblage; max frugivore size (extinct) = 95th percentile maximum body mass of extinct frugivores in the assemblage.

To disentangle the direct and indirect relationships between the distributions of frugivore body masses, human footprint, and plant seed widths in Madagascar, we used structural equation models (SEM) as implemented in the R package ‘lavaan’ v0.6–12 (Rosseel 2012) in R 4.5.0 (R Core Team 2025). We defined the a priori model structure to reflect the hypothesised relationships (Figure 1). First, the model included the potential ‘downsizing’ effect of human footprint on maximum plant seed width, directly and indirectly via ‘downsizing’ maximum extant frugivore body mass. Moreover, the ‘legacy’ effect of extinct megafrugivores was modelled by testing for a relationship between assemblage maximum extinct frugivore body mass and maximum plant seed width. Furthermore, we included the effects of environmental PC1 and PC2 on plant seed width, extant and extinct frugivore body mass, and human footprint. We allowed covariance between human footprint and maximum extinct frugivore body mass, and between maximum body masses of extant and extinct frugivores. By allowing the residual variances of two observed variables to be correlated, we acknowledge that these variables may share underlying historical or environmental drivers that are not explicitly included in the model. We log‐transformed biological variables (i.e., seed widths and body masses) to approach normal distributions of model residuals, and scaled all variables to 0–1 to compare standardised effect sizes among predictor variables. We used the ‘MLR’ estimator when conducting the SEM, which applied data‐based corrections to the test statistics and standard errors to offset the bias introduced by the non‐normal distribution of model residuals (Rosseel 2012).

We fitted three SEMs to the data. First, we fitted a SEM using all 361 assemblages (30 km grid; similarly applied to the other two grid sizes) to identify direct and indirect effects of human footprint on seed size (H1). Then, to evaluate the effect of past interactions on seed size of anachronistic mega‐seeded plants (H2), we used a comparative framework, in which we compared the effects of the predictor variables for mega‐seeded and non‐mega‐seeded plants. To this end, we fitted the same a priori SEM with two subsets of the data: assemblages with mega‐seeded plants (66 assemblages) and assemblages without mega‐seeded plants (295 assemblages) (Figure S1).

We fitted the SEM models separately to each of the 20 imputed datasets as well as the dataset without imputation. We pooled the results from the 20 imputed datasets using Rubin's rules (Rubin 1987), implemented via the ‘lavaan.mi’ R package v0.1–0 (Jorgensen 2025). To refine the model structure, we followed a stepwise removal of statistically non‐significant paths starting from the a priori model. We excluded the path with the highest *p*‐value from the model, reran the SEM, and repeated this procedure until only paths representing a significant effect remained. Following Hooper et al. (2008), since different indices capture different aspects of model fit, we considered a final model a good fit if a combination of conditions was met: the root mean square error of approximation (RMSEA) smaller than 0.05, the comparative fit index (CFI) greater than 0.95, and the Chi‐square test not significant (*p* > 0.05).

### 2.6 Randomisations and Spatial Autocorrelation

To assure that relationships revealed in the final SEM models deviated from random expectations, we reran the final SEM on 1000 randomised seed width datasets and compared the resulting null distributions with the observed effect sizes and *p*‐values. For more details, see Supporting Information Extended Methods S1.

Significant effects recovered from the SEM analyses may be derived from spatial autocorrelation among assemblages. Therefore, we tested for spatial autocorrelation in our focal variable—maximum seed width—and conducted spatial autoregressive (SAR) modelling to correct for potential effects of spatial autocorrelation on outcomes. For more details, see Supporting Information Extended Methods S1.

## 3 Results

**FIGURE 2:**
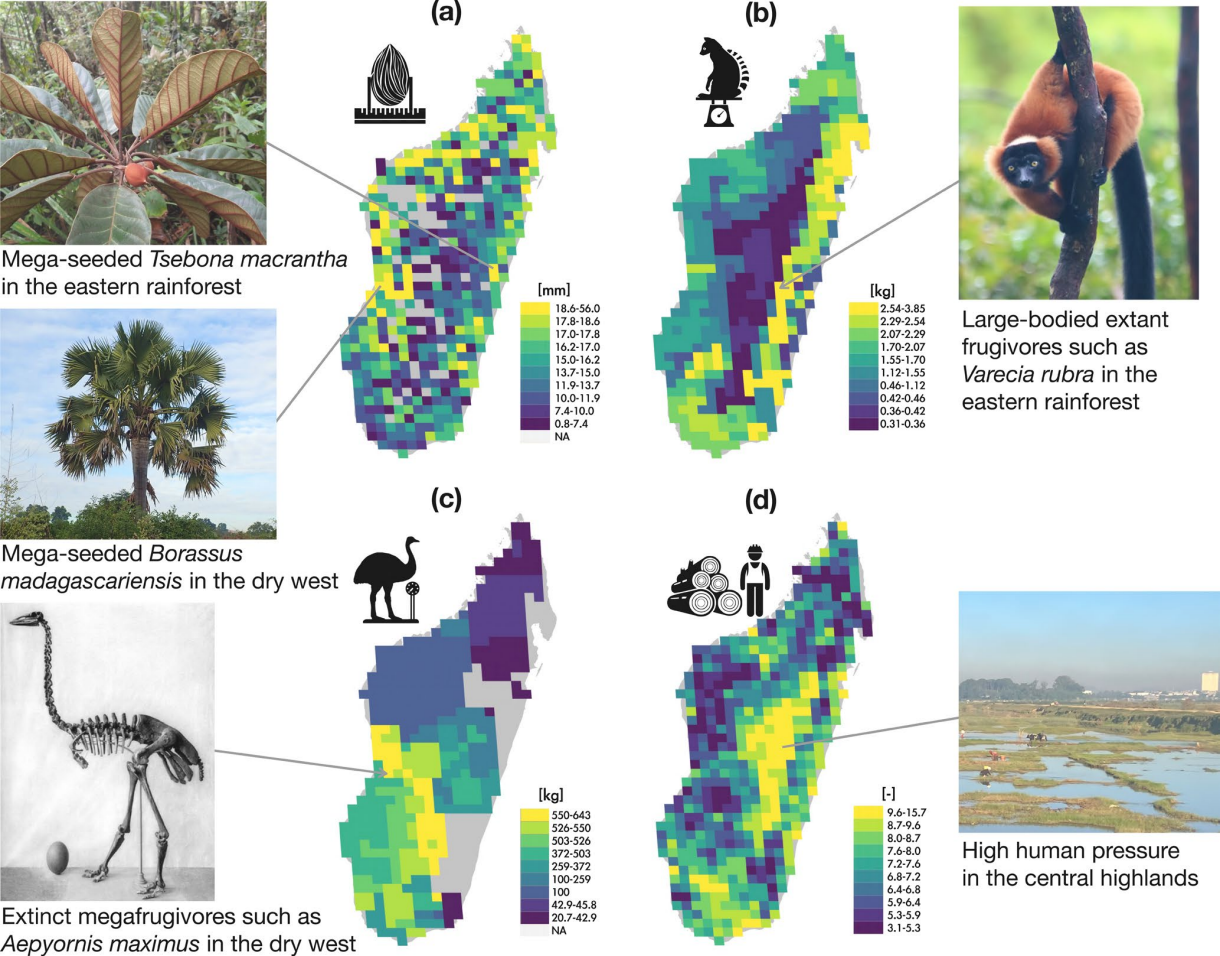
Distribution of seed size, extant and extinct frugivore size, and human footprint on Madagascar. These four main variables are used in the structural equation models. (a) 95th percentile maximum plant seed width in each assemblage; (b) 95th percentile maximum body mass of extant frugivores in each assemblage; (c) 95th percentile maximum body mass of extinct frugivores in each assemblage; (d) average human footprint of 2009 from Venter et al. (2016). Colour gradients of all variables are determined using quantile classification of unprocessed original values into ten classes. Photos of representative species with the largest seeds, largest frugivores, and area with the highest human footprint are shown alongside their locations on the maps. Photo credit: Aina Razanatsima (top left, downloaded from Madagascar Catalogue Image Gallery (http://legacy.tropicos.org/Image/100560806?projectid=17)); Laura Méndez (middle left); Monnier (bottom left, downloaded from https://commons.wikimedia.org/wiki/File:Aepyornis_maximus.jpg); Liuye (top right, downloaded from iNaturalist (https://www.inaturalist.org/observations/249596226)); Yuanshu Pu (bottom right).

Seed sizes, extant and extinct frugivore body masses, and human footprint distinctly varied across Madagascar (Figure 2). Assemblages in the eastern rainforests and the western drylands had larger maximum seed widths than assemblages in the central highlands and the southern spiny thickets (Figure 2a). Extant frugivores with the largest body masses were concentrated in the eastern rainforests (Figure 2b), whereas extinct frugivores with the largest body masses were primarily distributed in the dry west (Figure 2c). Finally, human footprint was highest in the central highlands around major cities such as Antananarivo and Fianarantsoa, as well as in southern and eastern coastal areas (Figure 2d).

### 3.1 PCA of Environmental Factors

The first two principal components together accounted for 71.25% variance in all environmental factors across assemblages (Figure S2). PC1 (39.63% variance) reflected a seasonal drought gradient (dry to wet), that is, positive values were associated with less climatic water deficit, lower evapotranspiration, lower temperature in the warmest months, more precipitation in the driest months, lower precipitation seasonality, and fewer dry months. PC2 (31.62% variance) reflected a gradient from central highland savannas to lowland environments, that is, positive values were associated with lower temperature seasonality, higher temperature in the coldest months, more annual precipitation, narrower temperature ranges, and lower altitude. By using these two PC axes in the SEMs, we accounted for the effects of environmental variation on our focal variables (i.e., plant seed width, extant and extinct frugivore body mass and human footprint).

### 3.2 Predictors of Maximum Seed Width

**FIGURE 3:**
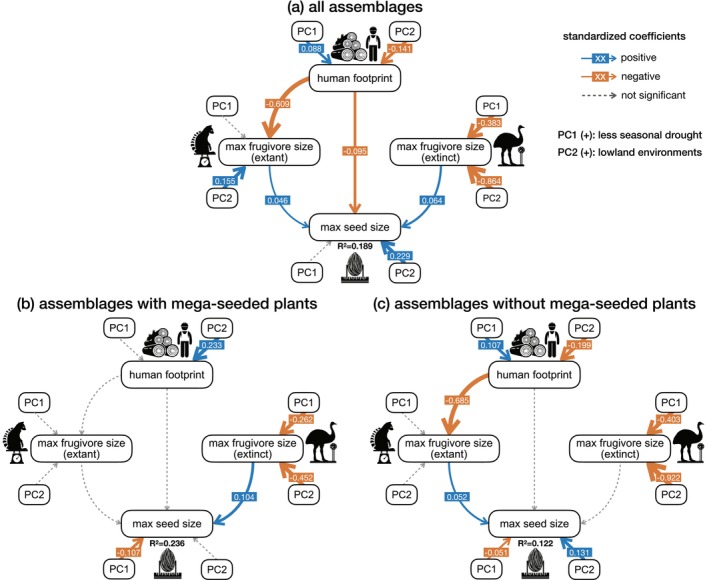
Path diagram showing the relationships between seed size, frugivore size, human footprint and environmental factors in Malagasy plant assemblages. The best fitting structural equation models after model selection are shown, using (a) the Madagascar‐wide dataset of 361 assemblages with at least 50% terrestrial area, three endozoochorous plant species, three extant frugivore species and one extinct frugivore species; (b) a subset of 66 assemblages where mega‐seeded plants occur (i.e., plants with seeds that are too large to be dispersed by any extant Malagasy frugivore); (c) a subset of 295 assemblages where no mega‐seeded plants occur. Model (a) tests Hypothesis 1 (‘downsizing‐effect’ across all assemblages), while model (b) and (c) together test Hypothesis 2 (comparing ‘legacy‐effect’ between assemblages with and without mega‐seeded plants). Effects of the first two principal components of environmental factors on seed size, frugivore size, and human footprint are shown next to each variable. Covariances allowed in the model are omitted in the figure. The marginal coefficients of determination (*R* ^2^) are given for the focal response variable, max seed size. Solid arrows indicate significant effects; dashed arrows indicate insignificant effects. The effect sizes of significant standardised coefficients are specified on the arrows, corresponding to the thickness of the arrows (blue = positive effects; orange = negative effects). Max seed size = 95th percentile maximum seed width in the assemblage; max frugivore size (extant) = 95th percentile maximum body mass of extant frugivores in the assemblage; max frugivore size (extinct) = 95th percentile maximum body mass of extinct frugivores in the assemblage.

In the full SEM model testing H1, we found significant effects of all focal predictors on maximum seed width among the 30 × 30 km assemblages with a good model fit (Figure 3a, see full SEM fit indices in Table S5). Specifically, the human footprint had a relatively weak direct negative effect on maximum seed width and a relatively strong indirect negative effect via frugivore downsizing, that is, decreasing maximum body mass of extant frugivores. In addition, maximum body masses of both extant and extinct frugivores had relatively weak positive effects on maximum seed widths, indicating that seeds are larger in assemblages with larger extant or extinct frugivores. Environmental factors influenced all variables. Specifically, larger seeds and larger extant frugivores were found in lowland environments (PC2). Larger extinct megafrugivores were found in areas that nowadays have more seasonal drought (PC1) and in the central highlands (PC2). Areas with less extreme drought (PC1) and central highlands (PC2) had a higher human footprint. Together, 18.9% variation in maximum seed width was explained by the full SEM.

When analysing assemblages with and without mega‐seeded plants separately to test H2, distinct effects of extant and extinct frugivores were revealed. Specifically, in assemblages with mega‐seeded plants (Figure 3b), maximum seed width was only significantly explained by the maximum body mass of extinct megafrugivores, not extant frugivores. In contrast, in assemblages without mega‐seeded plants (Figure 3c), maximum seed width was only significantly explained by the maximum body mass of extant frugivores, not extinct ones. Additionally, in both subsets, larger seed width was associated with more seasonal drought (PC1). In assemblages without mega‐seeds, larger seed width was also associated with lowland environments (PC2). No environmental effects on maximum extant frugivore body masses were detected, but in both subsets, larger maximum body masses of extinct frugivores were found under more seasonal drought (PC1) and in the central highlands (PC2), consistent with the full SEM model. Our predictors explained 23.6% variation in maximum seed width in assemblages with mega‐seeded plants, and 12.2% in assemblages without mega‐seeded plants.

### 3.3 Sensitivity Analyses

In the sensitivity analysis using only directly measured seed widths without imputed data, we found very similar results to the analysis that used the full dataset (Figure S3; see more details in the Supporting Information Extended Results S2).

In the sensitivity analysis testing the robustness of our results to different grid sizes, all three SEMs acquired similar results with good model fit but smaller *R* ^2^ when using the smaller grid size (Figure S4; see more details in the Supporting Information Extended Results S2). However, when using the larger grid size, the direct downsizing effect of human footprint on seed width was not supported, and the model testing for H2—using assemblages with mega‐seeded plants only—did not reach a good fit (Figure S5; see more details in the Supporting Information Extended Results S2).

The simulations of assemblages with randomised seed width confirmed that relationships revealed from empirical data strongly deviated from the null scenario in which maximum seed widths were randomly distributed across assemblages in Madagascar (Table S6; see more details in the Supporting Information Extended Results S3).

Spatial autocorrelation was present in the Madagascar‐wide analysis, but not in the subset analyses. Furthermore, after correcting for spatial autocorrelation in the SAR model, key predictors of seed width remained robust, indicating that spatial autocorrelation did not substantially impact our main results (Table S7; see more detailsin the Supporting Information Extended Results S3).

## 4 Discussion

We found that the human footprint was negatively associated with the maximum seed width of an assemblage, both directly and indirectly via its association with reduced maximum extant frugivore body mass, supporting the downsizing‐effect hypothesis (H1, Figure 3a). Furthermore, our results illustrate that seed widths of anachronistic mega‐seeded species are explained by the past co‐occurrence with now extinct megafrugivores, whereas seed widths of smaller‐seeded species are explained by co‐occurrence with extant frugivores, thus supporting the legacy‐effect hypothesis (H2, Figure 3b,c).

Our results suggest that human pressure may be linked to the extirpation of large‐seeded plants via two mechanisms. First, we detected an indirect relationship between human footprint and seed width, via plant‐to‐frugivore trait matching. This evidences the cascading effects of human‐driven defaunation and ‘downsizing’ of animal assemblages on plants (Young et al. 2016). Specifically, frugivore downsizing alters key functional aspects of seed dispersal, such as reducing dispersal distances, and numbers and sizes of seeds being dispersed (Donoso et al. 2020; Markl et al. 2012; Pérez‐Méndez et al. 2016). It also affects short‐term population recruitment, for example increasing seedling aggregation and reducing recruitment of large‐seeded plants (Ganzhorn et al. 1999; Harrison et al. 2013; Kurten 2013; Razafindratsima et al. 2021). Finally, for longer‐term outcomes at the community level, defaunation leads to losses in species richness and diversity (Donoso et al. 2020; Kurten 2013). Our trait‐based study adds to this growing literature by revealing size‐selected changes in specific trait structures of plant assemblages associated with frugivore downsizing and dispersal limitation. Importantly, our structural equation models do not elucidate the mechanisms by which such changes may happen. Possibly, the loss of large‐bodied frugivores leads to secondary extinctions, hence the extirpation of large‐seeded species. Alternatively, large‐seeded species can evolve smaller seeds in adaptation to the remnant frugivore community. Such adaptation can happen rapidly, sometimes within decades, as shown in the palm *Euterpe edulis* in the Atlantic Forest of Brazil (Galetti et al. 2013).

Second, via a direct path, human footprint was negatively associated with assemblage‐level maximum seed widths. This suggests that larger‐seeded plants may be particularly vulnerable to higher human pressure, or conversely, that smaller‐seeded plants are favoured under higher human pressure. However, this direct downsizing effect was not supported in some of the sensitivity analyses (i.e., without imputed data and with larger grid cells; Figures S3a and S5a), whereas the indirect downsizing effect through the dispersal‐related pathway, that is, frugivore downsizing, is more prominent and robust to data imputation and different grid sizes (Figures S3a, S4a, S5a).

Assemblage‐level seed downsizing can further alter forest structure and ecosystem function, because seed size is allometrically related to many key plant functional traits, such as plant height, diameter at breast height, and wood density (Hawes et al. 2020; Moles et al. 2005; Razafindratsima, Gentles, et al. 2018). These traits are associated with important ecological functions such as the potential carbon storage capacity of a species (Chave et al. 2005). Hence, assemblage‐level seed downsizing may ultimately have destructive consequences for the carbon storage of tropical forests as a whole (Bello et al. 2015; Gardner et al. 2019; Razafindratsima, Yacoby, and Park 2018).

Additionally, the abiotic environment plays an important role in modulating the size‐selective changes in trait structures of plant and frugivore assemblages (Figure 3). Larger seeds tend to occur in lowland rainforests or more drought‐prone habitats (Figures 2a, 3), consistent with theories that large seeds increase survival in shaded forest environments (Leishman and Westoby 1994) and improve drought/stress tolerance (Westoby et al. 1996; Wölke et al. 2023). Similarly, larger extant frugivores are found in humid lowland environments (Figures 2b, 3), indicating that the present‐day largest frugivorous lemurs depend on rainforest habitats. Although the current human footprint remains relatively low in these areas (Figures 2d, 3), projected forest loss in Madagascar shows that, even in the absence of human impact, these rainforest habitats may decline by 62% solely due to climate change (Morelli et al. 2020).

With overall larger seeds found in assemblages with larger frugivores, the global trend of plant‐frugivore trait matching observed in previous studies holds at finer spatial extents in Madagascar (Durand‐Bessart et al. 2023; Lim et al. 2020; McFadden et al. 2022). However, we acknowledge that our model only explained a small proportion of the variation in seed size (*R* ^2^ = ca. 20%). This may be partly due to a mismatch in spatial resolution between the occurrence‐based distribution of plants and the polygon‐based distribution of frugivores, potentially limiting the model's explanatory power. Supporting this, sensitivity analyses show that a smaller grid size further reduces the variance explained in seed width, while a larger grid size increases the *R* ^2^ but reduces the number of assemblages, thereby reducing statistical power in a different way. This suggests that spatial scale influences the observation and quantification of ecological relationships and calls for careful consideration in study design. Additionally, our approach assumes interactions between co‐occurring species that may not actually interact. Therefore, incorporating interaction‐based data or other key traits, such as the degree of frugivory, may better capture the complexity of dispersal interactions and explain more variation in seed size distribution (Guerra et al. 2025). For example, we excluded facultative frugivores in our study, but some of these species—such as small‐bodied omnivores like *Microcebus spp* and some tortoises like *Pyxis planicauda* —can play key roles in seed dispersal in degraded forests and dry or transitional ecosystems, where strict frugivores ‐ with > 50% fruit in diet ‐ are scarce.

Although our model predicts seed downsizing following frugivore downsizing, empirical data illustrate that 15 mega‐seeded plant species have persisted in Madagascar since the extinction of their primary dispersers—the Quaternary megafrugivores (Table 1). These plants seem to have persisted in their historical distribution ranges, with larger seeds found in places with larger past megafrugivores (Figure 3b). For example, in the dry deciduous forest and the succulent woodlands in west Madagascar, species such as *Borassus madagascariensis* (Arecaceae) and *Adansonia suarezensis* (Malvaceae) bear the largest seeds and fruits in Madagascar (Figure 2a; seed width up to 67.5 mm wide), possibly once dispersed by co‐existing megafauna such as the elephant birds (Figure 2a,c; Albert‐Daviaud et al. 2020; Janzen and Martin 1982; Méndez et al. 2022). Here, while the comparative body mass distribution pattern of extinct frugivores supports the hypothesised legacy effect on the mega‐seeded plants, the geographic extent of this relationship should be interpreted with caution. This is because the reconstructed distributions of extinct megafrugivores—though adjusted for sampling bias using coeval fossil species—remain susceptible to spatial bias from uneven fossilisation probabilities and shifting co‐occurring patterns across fossil species (Carotenuto et al. 2020).

It remains unclear how these mega‐seeded plants have persisted. They may have found alternative dispersal agents, such as introduced zebu cattle, bushpigs, humans, or water (Andrianjakarivelo 2003; Andriantsaralaza et al. 2024; Guimarães et al. 2008; Méndez et al. 2024; Tonos et al. 2024). Hence, dispersal dysfunctionality may not be a major threat. Alternatively, their long life spans and generation times may have allowed them to persist, despite potentially shrinking range sizes and reductions in effective population sizes, as observed, for example, on the Canary islands (Pérez‐Méndez et al. 2016) and in South America (Doughty et al. 2016; Petrocelli et al. 2024). Possibly, many mega‐seeded species have already disappeared since the extinction of the megafauna, although direct evidence from fossil records is scarce, especially in rainforests. At a broader time and spatial scale, increased extinction rates of mega‐seeded palms and transition rates to smaller seeds in the Quaternary (last 2.6 million years) hint that seed downsizing may already be in a progressing stage (Onstein et al. 2018).

In conclusion, we applied a causal framework to examine potential direct and indirect downsizing effects of human pressure on seed size in Madagascar. Our findings emphasise the urgent need for conservation efforts in areas with increasing human pressure (Semper‐Pascual et al. 2023) and that conservation prioritisations should take species interactions and trait‐based ecology into consideration (Falcón and Hansen 2018; Ganzhorn et al. 2024). Furthermore, the distribution of mega‐seeded plants holds exclusive signatures of past co‐occurring but now extinct megafaunal frugivores, evidencing how current biodiversity patterns cannot be fully understood without considering the (recent) past (Méndez et al. 2022, 2024; Xu et al. 2023). To forecast the future of plant assemblages under continuing frugivore downsizing, further research is needed to better understand how remnant mega‐seeded species have persisted in ecosystems without their primary seed dispersers.

## Author Contributions

Y.P. and R.E.O. developed the ideas, hypotheses and methodology for the study. Y.P. and A.Z. collected and curated the data. Y.P. performed the analyses. R.E.O. and A.Z. provided critical comments and interpretations during analyses. Y.P. and R.E.O. wrote the original manuscript. All authors reviewed and edited the manuscript. R.E.O. supervised the project.

## Peer Review

The peer review history for this article is available at https://www.webofscience.com/api/gateway/wos/peer‐review/10.1111/ele.70205.

## Supporting information

Supporting_Information.pdf: including Extended Methods S1, Extended Results S2‐S3, Supporting Figures S1‐S5, and Supporting Tables S1‐S7

## Data Availability

All raw species‐level data, compiled assemblage‐level data, and R scripts used for data processing and analyses are publicly available in the Dryad Digital Repository (DOI: https://doi.org/10.5061/dryad.pvmcvdnx7). The repository also includes version information of all R packages used in the scripts and a detailed README file to facilitate full reproducibility.
