# Supplementary material for: Legacy of the Lost and Pressure of the Present: Malagasy Plant Seeds Retain Megafauna Dispersal Signatures but Downsize Under Human Pressure
Source: Ecol Lett. 2025 Sep 10;28(9):e70205. doi: 10.1111/ele.70205 (PMC12423571; doi:10.1111/ele.70205)
Supplement: Supplementary file 1 — Supporting_Information.pdf: including Extended Methods S1, Extended Results S2‐S3, Supporting Figures S1‐S5, and Supporting Tables S1‐S7 [file ELE-28-0-s001.pdf]

Supporting Information for

Legacy of the lost and pressure of the present: Malagasy plant seeds retain megafauna dispersal signatures but downsize under human pressure

Yuanshu Pu[1,2], Alexander Zizka[3,4], Renske E. Onstein[4,5,1,2]

[1]University of Leipzig, Faculty of Life Sciences, Leipzig, DE
[2]German Centre for Integrative Biodiversity Research (iDiv) Halle-Jena-Leipzig, Leipzig, DE
[3]Department of Biology, Philipps-University Marburg, Marburg, DE
[4]Naturalis Biodiversity Centre, Leiden, NL
[5]Leiden University, Institute of Biology Leiden, Leiden, NL

Yuanshu Pu: yuanshu.pu@evobio.eu
Renske E. Onstein: renske.onstein@naturalis.nl
Alexander Zizka: alexander.zizka@biologie.uni-marburg.de

Corresponding author:
Yuanshu Pu
Faculty of Life Sciences, University of Leipzig
Talstraße 33
04103 Leipzig, Germany
Phone: +49 15257550274
E-mail: yuanshu.pu@evobio.eu

This document includes

- Extended Methods S1
- Extended Results S2-S3
- Supporting Figures S1-S5
- Supporting Tables S1-S7

Extended Methods S1: Randomisations and spatial autocorrelation

To assure that relationships revealed in the final SEM models deviated from random expectations, we reran the final SEM after randomizing maximum seed widths across assemblages, and repeated this 1,000 times. We obtained p-values and effect sizes for each predictor of maximum seed width in the randomized scenarios, and assessed whether observed effect sizes and p-values from the empirical data significantly differed (i.e., outside 95% distribution) from randomized inferences.

To test for spatial autocorrelation in our focal variable – maximum seed width – and to correct for its effects using spatial autoregressive (SAR) modelling, we used the R package "spdep" v1.2-5 (Bivand & Wong, 2018). First, we defined the spatial weight matrix for the SAR models, using a minimum distance that can link each assemblage grid to at least one other assemblage grid (i.e., 44.6 km). Next, we evaluated the estimate and significance of all direct predictor variables on maximum seed widths that were identified as significant in the SEM, in a non-spatial linear regression model and a SAR model, which were directly comparable, as they only differed in their correction for spatial autocorrelation. Finally, we calculated Moran's I values for the linear model, the residuals of the linear model, and the SAR model, to evaluate the role of spatial autocorrelation in our data and model outcomes.

Extended Results S2: Imputation and grid size sensitivity

In the sensitivity analysis using only directly measured seed widths without imputed data, 3 out of 361 assemblages had to be excluded because their 95 percentile maximum seed widths became NA, as none of the species had measured seed width data. Nevertheless, we found very similar results as when using the full imputed seed width dataset, with similar effect sizes and significance of drivers of seed width (Fig. S3, Table S5), except that the direct effect of human footprint on maximum seed width became insignificant with $p = 0.089$ (Fig. S3a).

When testing the robustness of our results to different grid sizes, the focal relationships remained similar when using a smaller grid size, but showed some discrepancy when using a larger grid size. Specifically, when using 15 km × 15 km grid size, all three SEMs acquired similar results with good model fit, but smaller R squares than models using 30 km x 30 km grid size (Fig. S4). Only the

relationship between maximum body mass of extinct frugivores and maximum seed width was insignificant when including all assemblages in Madagascar (Fig. S4a). However, it remained significant among assemblages with mega-seeded plants (Fig. S4b). When using 45 km × 45 km grid size, the indirect downsizing effect of human footprint on maximum seed width remained significant, but the direct effects of human footprint and extinct frugivore body mass on seed width became insignificant. The model testing H1, using all assemblages (Fig. S5a) and the model testing H2, using assemblages without mega-seeded plants (Fig. S5c), reached good model fit with larger R squares than models using 30 km x 30 km grid size. However, when analysing assemblages with mega-seeded plants only (Fig. S5b, Table. S5), the model did not reach a good fit, which may relate to the low number of observations (48) relative to the number of model parameters (12; Wolf et al., 2013).

Extended Results S3: Randomisations and spatial autocorrelation

In the 1,000 simulations with randomised maximum seed widths across assemblages, all predictors of seed width became insignificant in ca. 95% of the simulations (see Table S6 for detailed values). Moreover, effect sizes from the empirical data were larger than 95% of the simulated effect sizes for all predictors (Table S6). This emphasizes that relationships revealed from empirical data strongly deviated from a scenario in which maximum seed widths were randomly distributed across assemblages in Madagascar.

Spatial autocorrelation was detected for the residuals in the drivers of maximum seed width using the Madagascar-wide dataset, but not when analysing assemblages with and without mega-seeded plants separately (Table S7). The SAR model for the Madagascar-wide dataset corrected for the spatial autocorrelation in the data, as evidenced by Moran's I tests (Table S7). Moreover, in the SAR model, all predictors of maximum seed width remained significant (at $P < 0.05$), but the effect of maximum extant frugivore body mass on maximum seed width was only marginally significant ($P = 0.055$). All predictors had similar standardized coefficients as in the non-spatial linear regression model (Table S7). This suggests that spatial autocorrelation did not substantially impact our main findings and conclusions.

Supporting Figures:

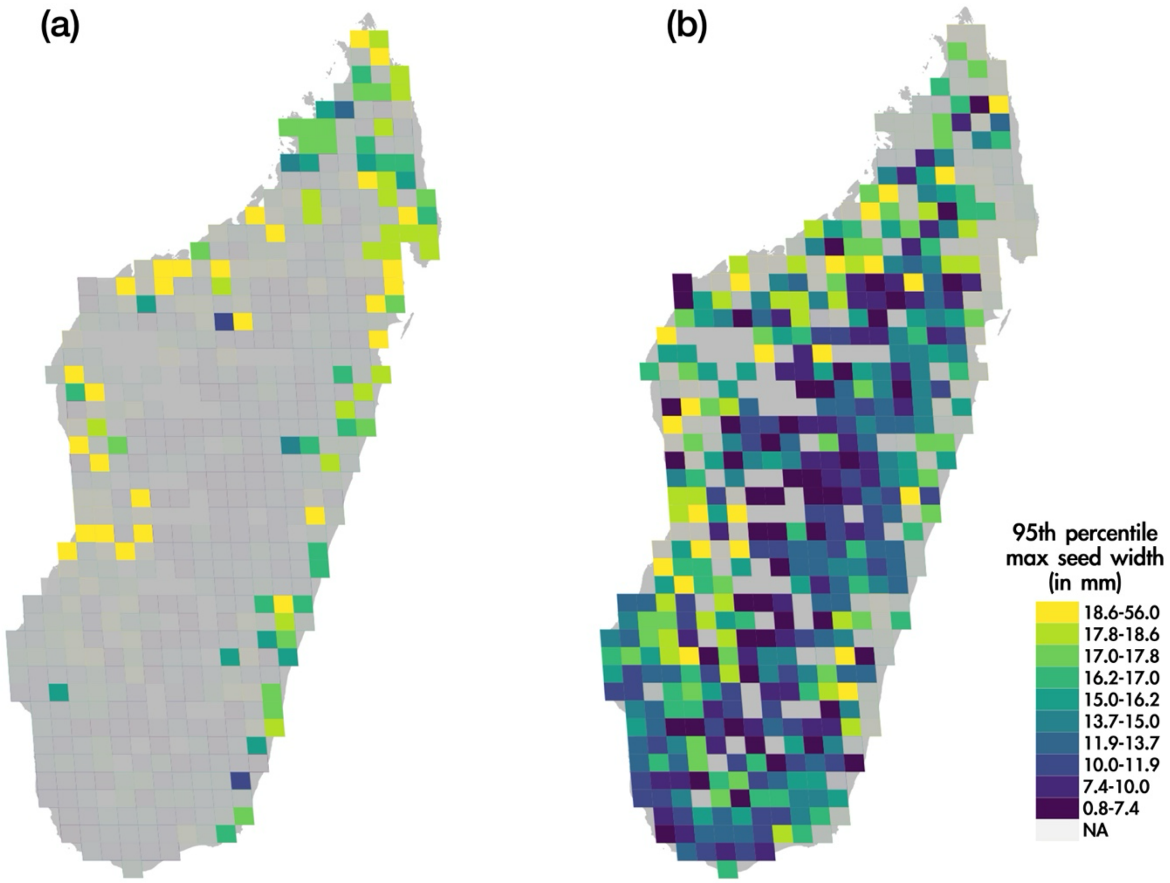

Figure S1: Distribution maps of the two subsets of Malagasy endozoochorous plant assemblages (30 km x 30 km). a) assemblages with mega-seeded plant species; b) assemblages without mega-seeded plant species. Species with seeds wider than 24.6 mm, the largest seed width dispersed by extant frugivores in Madagascar, are considered as mega-seeded plants. Assemblages that do not belong to each subset are shaded in grey. The colour gradient represents the 95th percentile maximum seed width of each assemblage, and is determined using quantile classification of unprocessed original values into ten classes.

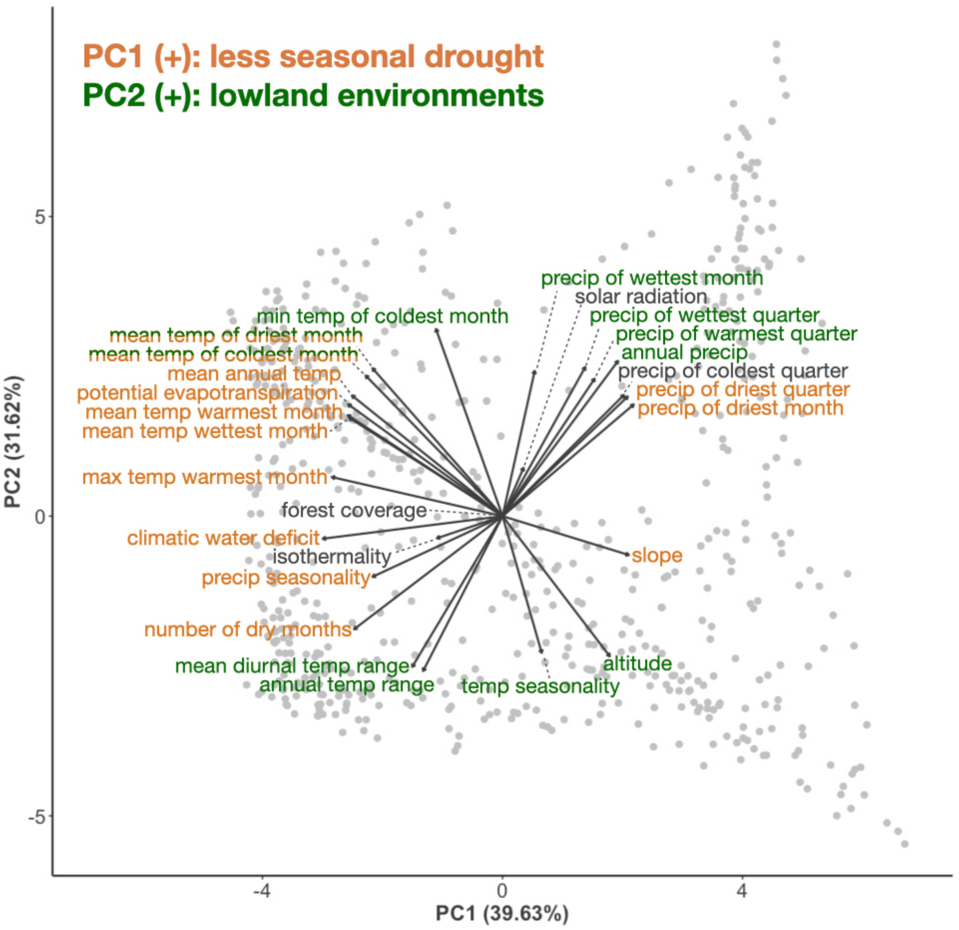

Figure S2: Principal Component Analysis (PCA) of environmental variables across assemblages in Madagascar. Distribution of the first two principal components (PC) are shown. Each dot in the scatter plot corresponds to one of 649 assemblages. Each arrow corresponds to the loadings of an environmental variable. The environmental variables with the highest contributions to PC1 (loadings larger than 0.2) are highlighted in orange, as is the summary of their associated environmental gradient. Similarly, the highest contributing variables (loadings larger than 0.2) and associated environmental gradient for PC2 are highlighted in green.

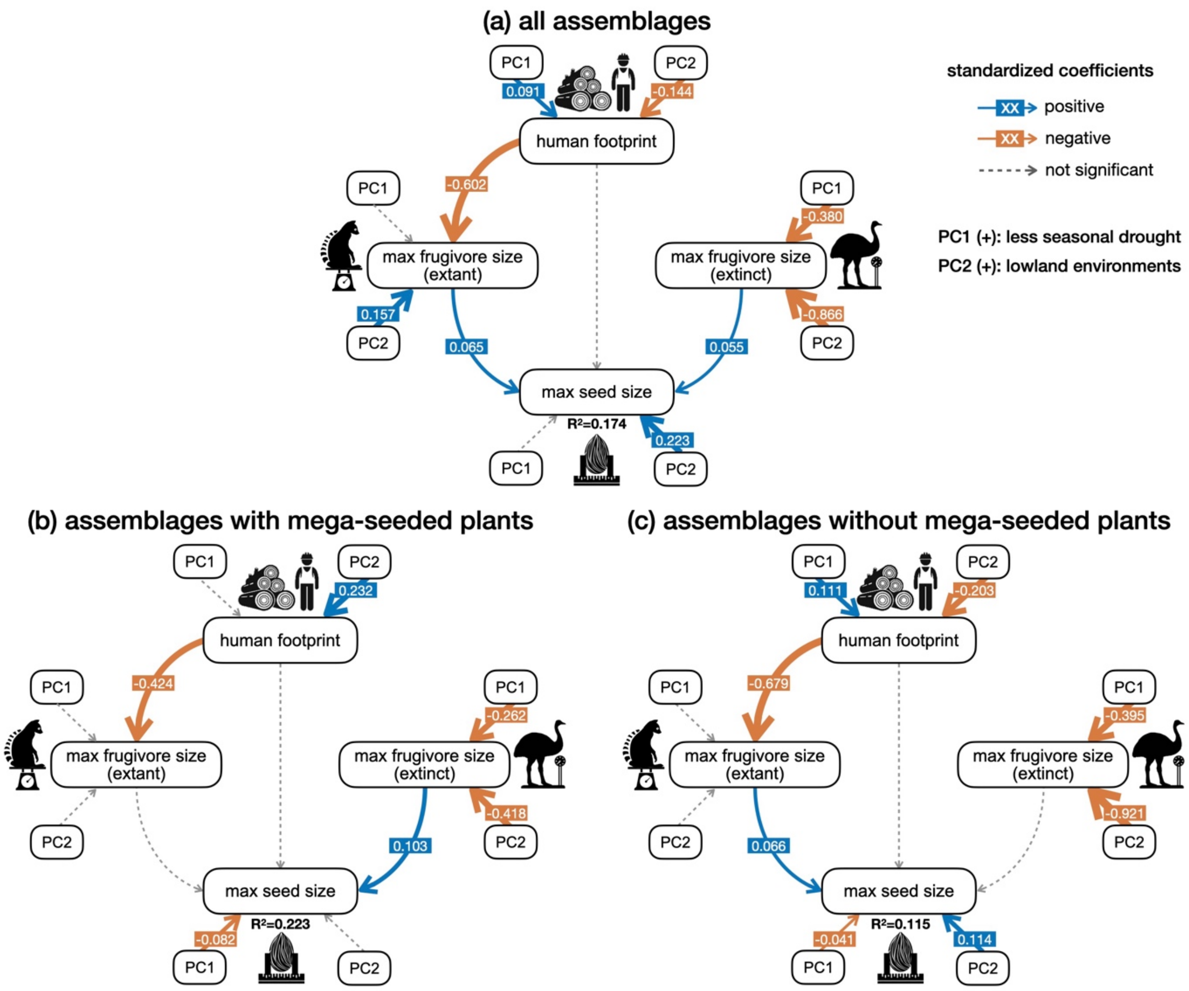

Figure S3: Path diagram showing the relationships between seed size, frugivore size, human footprint, and environmental factors in Malagasy plant assemblages, using only directly measured seed width without imputed data. The best fitting structural equation models after model selection are shown, using (a) the Madagascar-wide dataset of 358 assemblages with at least 50% terrestrial area, three endozoochorous plant species, three extant frugivore species and one extinct frugivore species; (b) a subset of 66 assemblages where mega-seeded plants occur (i.e., plants with seeds that are too large to be dispersed by any extant Malagasy frugivore); (c) a subset of 292 assemblages where no mega-seeded plants occur. Effects of the first two principal components of environmental factors on seed size, frugivore size, and human footprint are shown next to each variable. Covariances allowed in the model are omitted in the figure. The marginal coefficients of determination (R^2) are given for the focal response variable, max seed size. Solid arrows indicate significant effects; dashed arrows indicate insignificant effects. The effect sizes of significant standardized coefficients are specified on the arrows, corresponding to the thickness of the arrows (blue = positive effects; orange = negative effects). Max seed size = 95th percentile maximum seed width in the assemblage; max frugivore size (extant) = 95th percentile maximum body mass of extant frugivores in the assemblage; max frugivore size (extinct) = 95th percentile maximum body mass of extinct frugivores in the assemblage.

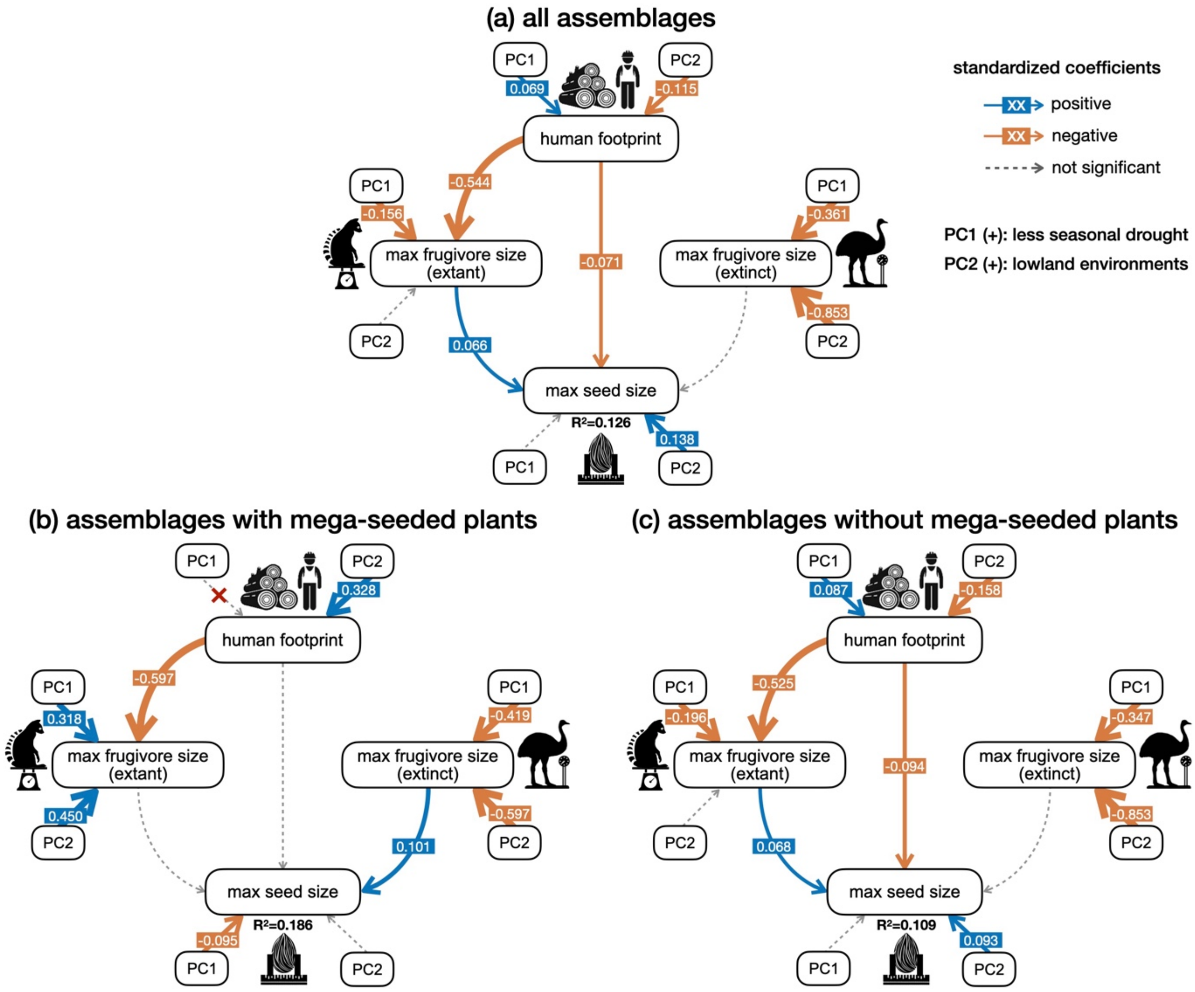

Figure S4: Path diagram showing the relationships between seed size, frugivore size, human footprint, and environmental factors in Malagasy plant assemblages, using 15 km x 15 km grid size. The best fitting structural equation models after model selection are shown, using (a) the Madagascar-wide dataset of 752 assemblages with at least 50% terrestrial area, three endozoochorous plant species, three extant frugivore species and one extinct frugivore species; (b) a subset of 88 assemblages where mega-seeded plants occur (i.e., plants with seeds that are too large to be dispersed by any extant Malagasy frugivore); (c) a subset of 664 assemblages where no mega-seeded plants occur. Effects of the first two principal components of environmental factors on seed size, frugivore size, and human footprint are shown next to each variable. Covariances allowed in the model are omitted in the figure. The marginal coefficients of determination (R^2) are given for the focal response variable, max seed size. Solid arrows indicate significant effects; dashed arrows indicate insignificant effects. The effect sizes of significant standardized coefficients are specified on the arrows, corresponding to the thickness of the arrows (blue = positive effects; orange = negative effects). Max seed size = 95th percentile maximum seed width in the assemblage; max frugivore size (extant) = 95th percentile maximum body mass of extant frugivores in the assemblage; max frugivore size (extinct) = 95th percentile maximum body mass of extinct frugivores in the assemblage.

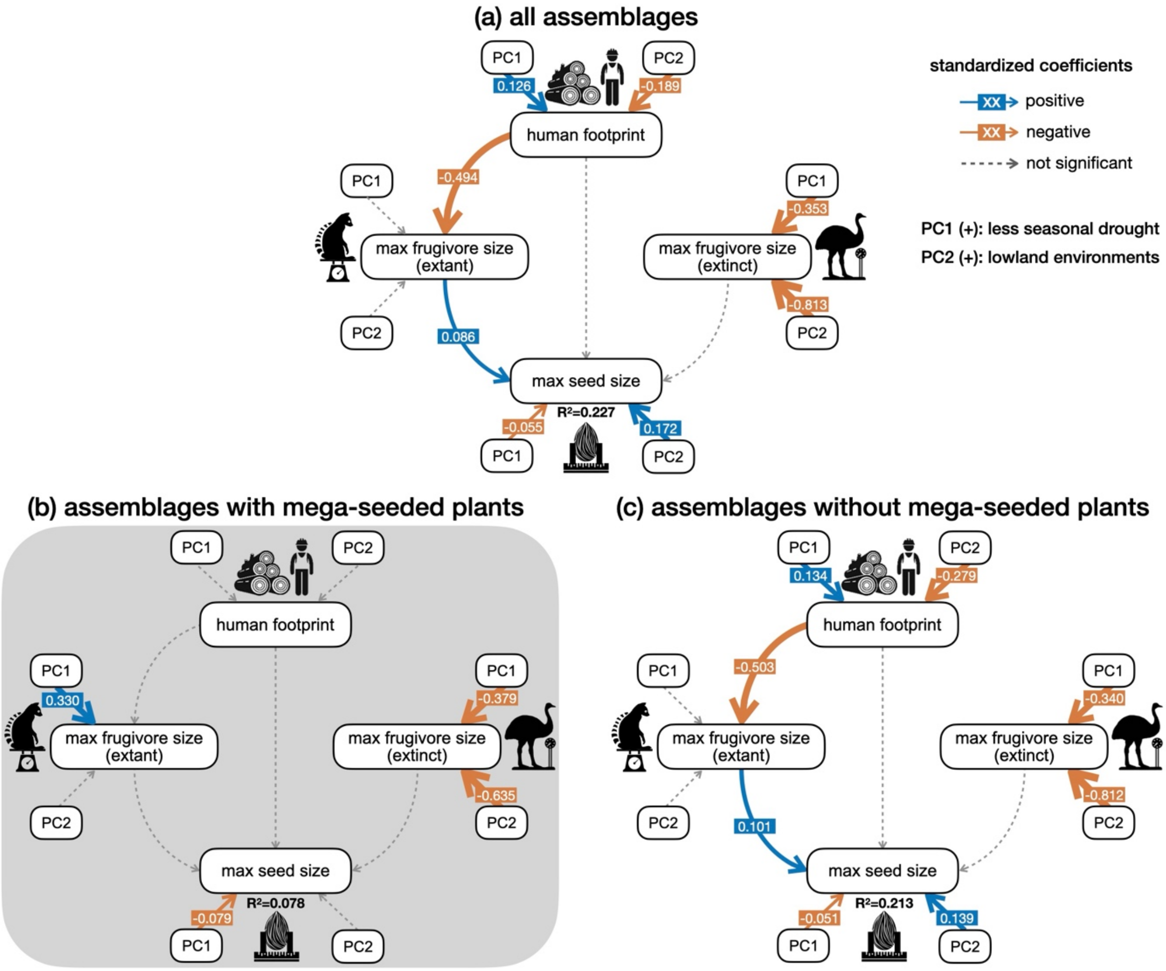

Figure S5: Path diagram showing the relationships between seed size, frugivore size, human footprint, and environmental factors in Malagasy plant assemblages, using 45 km x 45 km grid size. The best fitting structural equation models after model selection are shown, using (a) the Madagascar-wide dataset of 199 assemblages with at least 50% terrestrial area, three endozoochorous plant species, three extant frugivore species and one extinct frugivore species; (b) a subset of 48 assemblages where mega-seeded plants occur (i.e., plants with seeds that are too large to be dispersed by any extant Malagasy frugivore); (c) a subset of 151 assemblages where no mega-seeded plants occur. Grey background of panel (b) indicates that the model didn't reach a good fit (scaled RMSEA = 0.129, CFI = 0.855, Chi-Square test p-value = 0.095). Effects of the first two principal components of environmental factors on seed size, frugivore size, and human footprint are shown next to each variable. Covariances allowed in the model are omitted in the figure. The marginal coefficients of determination (R^2) are given for the focal response variable, max seed size. Solid arrows indicate significant effects; dashed arrows indicate insignificant effects. The effect sizes of significant standardized coefficients are specified on the arrows, corresponding to the thickness of the arrows (blue = positive effects; orange = negative effects). Max seed size = 95th percentile maximum seed width in the assemblage; max frugivore size (extant) = 95th percentile maximum body mass of extant frugivores in the assemblage; max frugivore (extinct) = 95th percentile maximum body mass of extinct frugivores in the assemblage.

Supporting tables:

Note: Table S1, S2, S3 are available in the Dryad Digital Repository (https://doi.org/10.5061/dryad.pvmcvdnx7)

Table S1. Taxonomy and fruit/seed traits of 3,241 endozoochorous plant species from Albert-Daviaud et al. (2020).

See file "Table S1 - 3241 endozoochorous plant species and fruit/seed traits.xlsx".

Column 1-3: species taxonomy

Column 4: whether there's filtered occurrence data from Ralimanana et al. (2022)

Column 5-7: original fruit and seed traits data collected by Albert-Daviaud et al. (2020)

Column 8-27: 20 independently imputed seed width datasets from Albert-Daviaud et al. (2020)

Table S2. Taxonomy, frugivory, and body masses of 48 extant frugivores and 15 extinct megafrugivores.

See file "Table S2 - frugivores species, frugivory and body masses.xlsx".

Sheet 1: 48 extant frugivores

- Column 1-5: species taxonomy
- Column 6: percentage of fruits in the diet of the species
- Column 7: reference for the source of frugivory data
- Column 8: body mass (in kg), averaged across available data from Wilman et al. (2014), Galán-Acedo et al. (2019) and Razafindratsima et al. (2018)

Sheet 2: 15 extinct megafrugivores

- Column 1-2: species taxonomy
- Column 3: body mass (in kg)
- Column 4: reference for the source of body mass data

Table S3. Species richness, largest seed widths, largest body masses, human footprint, and environmental data in 649 assemblages of plants and frugivores across Madagascar.

See file "Table S3 - assemblage-level data.xlsx".

Column 1: Assemblage ID
Column 2-3: Coordinates of the assemblage
Column 4: percentage of terrestrial area
Column 5: species richness of endozoochorous plant species
Column 6: species richness of extant frugivore species
Column 7: 95th percentile maximum body mass of extant frugivores (in kg, log-transformed)
Column 8: species richness of extinct frugivore species
Column 9: 95th percentile maximum body mass of extinct frugivores (in kg, log-transformed)
Column 10: averaged human footprint value (2009), adapted from Venter et al. (2016)
Column 11-12: the first two principal components of the principal component analysis (PCA) of 26 abiotic variables (for a complete overview of variables, see Table S4)
Column 13: whether at least one mega-seeded plant species occurs in the assemblage
Column 14-33: 20 versions of 95th percentile maximum plant seed width using 20 independently imputed seed width dataset (in mm, log-transformed)

Table S4. List of 26 environmental variables used in this study (source: MadaClim, https://madaclim.cirad.fr)

Category	Variable
Temperature	Annual mean temperature (°C)
	Mean diurnal range (mean of monthly (max temp - min temp)) (°C)
	Temperature annual range (BIO5-BIO6) (°C)
	Isothermality (mean diurnal range/temperature annual range x 100) (-)
	Temperature seasonality (standard deviation x 100) (°C)
	Max temperature of warmest month (°C)
	Min temperature of coldest month (°C)
	Mean temperature of wettest quarter (°C)
	Mean temperature of driest quarter (°C)
	Mean temperature of warmest quarter (°C)
	Mean temperature of coldest quarter (°C)
Precipitation	Annual total precipitation (mm)
	Precipitation seasonality (coefficient of variation) (-)
	Total precipitation of wettest month (mm)
	Total precipitation of driest month (mm)
	Total precipitation of wettest quarter (mm)
	Total precipitation of driest quarter (mm)
	Total precipitation of warmest quarter (mm)
	Total precipitation of coldest quarter (mm)
Water deficiency	Annual potential evapotranspiration (mm)
	Annual climatic water deficit (mm)
	Number of dry months in the year (-)
Geographic topology	Altitude (m)
	Slope (in degree)
-	Solar radiation (Wh.m-2.day-1)
-	Percentage of forest cover for the year 2010 (%)

Table S5. Summary of structural equation modelling final model fit indices.

Dataset		Number of observations	Indicies (scaled)		
			Chi-Square test	RMSEA	CFI
30 km x 30 km, with imputed data	all	361	0.397	0.000	1.000
	with megaseeds	66	0.347	0.041	0.980
	without megaseeds	295	0.263	0.035	0.994
30 km x 30 km, without imputed data	all	358	0.249	0.032	0.997
	with megaseeds	66	0.427	0.000	1.000
	without megaseeds	292	0.273	0.032	0.997
15 km x 15 km, with imputed data	all	752	0.278	0.020	0.999
	with megaseeds	88	0.671	0.000	1.000
	without megaseeds	664	0.366	0.006	1.000
45 km x 45 km, with imputed data	all	199	0.353	0.026	0.999
	with megaseeds*	48	0.095	0.129	0.855
	without megaseeds	151	0.319	0.039	0.996

Note: The p-value of model Chi-Square test, the root mean square error of approximation (RMSEA) and the comparative fit index (CFI) are shown for the best fitted models using each dataset. * indicates the model that didn't reach a good model fit (criteria: the Chi-Square test not significant ($p > 0.05$), RMSEA < 0.05, and CFI >0.95).

Table S6. Results of randomization simulations, using the full Madagascar-wide dataset (all 361 assemblages), the subset of 66 assemblages with mega-seeded plants, and the subset of 295 assemblages without mega-seeded plants.

Dataset	Effect	Frequency of significant effect in simulation	Observed effect size	95% confidence interval of effect size
Madagascar-wide	max plant seed width ~ max extant frugivore body mass	0.050	0.04	[-0.04,0.04]
	max plant seed width ~ max extinct frugivore body mass	0.043	0.08	[-0.05,0.05]
	max plant seed width ~ human footprint	0.055	-0.10	[-0.09,0.10]
with mega-seeds	max plant seed width ~ max extinct frugivore body mass	0.052	0.12	[-0.08,0.10]
without mega-seeds	max plant seed width ~ max extant frugivore body mass	0.048	0.05	[-0.04,0.04]

Note: Only predictors relevant to our three main hypotheses and significant in the SEM results were tested and shown. Column “Frequency of significant effect in simulation” indicates the proportions of runs out of 1000 simulations in which the effect was significant at $P < 0.05$.

Table S7. Results of Spatial autocorrelation test and Spatial autoregressive error model (SAR) for maximum seed width, in comparison with non-spatial linear regression model (LM).

Dataset	Moran's I of LM residual	Moran's I of SAR residual	Response	Predictors	Effect size in LM	Effect size in SAR
Madagascar-wide	0.085*	-0.003	max plant seed width	max extant frugivore body mass	0.041*	0.038 .
				max extinct frugivore body mass	0.077**	-0.076**
				human footprint	-0.103**	0.098*
				PC2	0.243**	0.243**
with mega-seeds	-0.21	-	-	-	-	-
without mega-seeds	0.064	-	-	-	-	-

Note: dataset “Madagascar-wide” refers to the full Madagascar-wide dataset (all 361 assemblages), “with mega-seeds” refers to the subset of 66 assemblages with mega-seeded plant, and “without mega-seeds” refers to the subset of 295 assemblages without mega-seeded plant species. Only significant predictors from the SEM results were tested and shown. ‘.’ indicates P values smaller than 0.06; ‘*’ indicates P values smaller than 0.05; ‘**’ indicates P values smaller than 0.01.

Supporting references:

Albert-Daviaud, A., Buerki, S., Onjalalaina, G. E., Perillo, S., Rabarijaona, R., Razafindratsima, O. H., Sato, H., Valenta, K., Wright, P. C., & Stuppy, W. (2020). The ghost fruits of Madagascar: Identifying dysfunctional seed dispersal in Madagascar's endemic flora. *Biological Conservation*, *242*, 108438. https://doi.org/10.1016/j.biocon.2020.108438

Bivand, R. S., & Wong, D. W. S. (2018). Comparing implementations of global and local indicators of spatial association. *TEST*, *27*(3), 716–748. https://doi.org/10.1007/s11749-018-0599-x

Galán-Acedo, C., Arroyo-Rodríguez, V., Andresen, E., & Arasa-Gisbert, R. (2019). Ecological traits of the world's primates. *Scientific Data*, *6*(1), 55. https://doi.org/10.1038/s41597-019-0059-9

Ralimanana, H., Perrigo, A. L., Smith, R. J., Borrell, J. S., Faurby, S., Rajaonah, M. T., Randriamboavonjy, T., Vorontsova, M. S., Cooke, R. S. C., Phelps, L. N., Sayol, F., Andela, N., Andermann, T., Andriamanohera, A. M., Andriambololonera, S., Bachman, S. P., Bacon, C. D., Baker, W. J., Belluardo, F., … Antonelli, A. (2022). Madagascar's extraordinary biodiversity: Threats and opportunities. *Science*, *378*(6623), eadf1466. https://doi.org/10.1126/science.adf1466

Razafindratsima, O. H., Yacoby, Y., & Park, D. S. (2018). MADA: Malagasy Animal trait Data Archive. *Ecology*, *99*(4), 990–990. https://doi.org/10.1002/ecy.2167

Venter, O., Sanderson, E. W., Magrach, A., Allan, J. R., Beher, J., Jones, K. R., Possingham, H. P., Laurance, W. F., Wood, P., Fekete, B. M., Levy, M. A., & Watson, J. E. M. (2016). Global terrestrial Human Footprint maps for 1993 and 2009. *Scientific Data*, *3*(1), 160067. https://doi.org/10.1038/sdata.2016.67

Wilman, H., Belmaker, J., Simpson, J., de la Rosa, C., Rivadeneira, M. M., & Jetz, W. (2014). EltonTraits 1.0: Species-level foraging attributes of the world's birds and mammals: *Ecological Archives* E095-178. *Ecology*, *95*(7), 2027–2027. https://doi.org/10.1890/13-1917.1

Wolf, E. J., Harrington, K. M., Clark, S. L., & Miller, M. W. (2013). Sample Size Requirements for Structural Equation Models: An Evaluation of Power, Bias, and Solution Propriety. Educational and Psychological

Measurement, 73(6), 913–934.
https://doi.org/10.1177/0013164413495237
